# Momentary Factors and Study Characteristics Associated With Participant Burden and Protocol Adherence: Ecological Momentary Assessment

**DOI:** 10.2196/49512

**Published:** 2024-04-24

**Authors:** Allan D Tate, Angela R Fertig, Junia N de Brito, Émilie M Ellis, Christopher Patrick Carr, Amanda Trofholz, Jerica M Berge

**Affiliations:** 1 Department of Epidemiology and Biostatistics College of Public Health University of Georgia Athens, GA United States; 2 Humphrey School of Public Affairs University of Minnesota Minneapolis, MN United States; 3 Department of Family Medicine and Community Health University of Minnesota Medical School University of Minnesota, Twin Cities Minneapolis, MN United States; 4 Department of Family Medicine and Adult and Child Center for Outcomes Research and Delivery Science University of Colorado Anschutz Medical Campus Aurora, CO United States

**Keywords:** adherence, burden, data quality, ecological momentary assessment, mental health, mHealth, mobile health, participant adherence, public health, stress, study design, survey burden, survey

## Abstract

**Background:**

Ecological momentary assessment (EMA) has become a popular mobile health study design to understand the lived experiences of dynamic environments. The numerous study design choices available to EMA researchers, however, may quickly increase participant burden and could affect overall adherence, which could limit the usability of the collected data.

**Objective:**

This study quantifies what study design, participant attributes, and momentary factors may affect self-reported burden and adherence.

**Methods:**

The EMA from the Phase 1 Family Matters Study (n=150 adult Black, Hmong, Latino or Latina, Native American, Somali, and White caregivers; n=1392 observation days) was examined to understand how participant self-reported survey burden was related to both design and momentary antecedents of adherence. The daily burden was measured by the question “Overall, how difficult was it for you to fill out the surveys today?” on a 5-item Likert scale (0=not at all and 4=extremely). Daily protocol adherence was defined as completing at least 2 signal-contingent surveys, 1 event-contingent survey, and 1 end-of-day survey each. Stress and mood were measured earlier in the day, sociodemographic and psychosocial characteristics were reported using a comprehensive cross-sectional survey, and EMA timestamps for weekends and weekdays were used to parameterize time-series models to evaluate prospective correlates of end-of-day study burden.

**Results:**

The burden was low at 1.2 (SD 1.14) indicating “a little” burden on average. Participants with elevated previous 30-day chronic stress levels (mean burden difference: 0.8; *P*=.04), 1 in 5 more immigrant households (*P*=.02), and the language primarily spoken in the home (*P*=.04; 3 in 20 more non-English–speaking households) were found to be population attributes of elevated moderate-high burden. Current and 1-day lagged nonadherence were correlated with elevated 0.39 and 0.36 burdens, respectively (*P*=.001), and the association decayed by the second day (β=0.08; *P*=.47). Unit increases in momentary antecedents, including daily depressed mood (*P*=.002) and across-day change in stress (*P*=.008), were positively associated with 0.15 and 0.07 higher end-of-day burdens after controlling for current-day adherence.

**Conclusions:**

The 8-day EMA implementation appeared to capture momentary sources of stress and depressed mood without substantial burden to a racially or ethnically diverse and immigrant or refugee sample of parents. Attention to sociodemographic attributes (eg, EMA in the primary language of the caregiver) was important for minimizing participant burden and improving data quality. Momentary stress and depressed mood were strong determinants of participant-experienced EMA burden and may affect adherence to mobile health study protocols. There were no strong indicators of EMA design attributes that created a persistent burden for caregivers. EMA stands to be an important observational design to address dynamic public health challenges related to human-environment interactions when the design is carefully tailored to the study population and to study research objectives.

## Introduction

### Overview

Ecological momentary assessment (EMA), a type of mobile health (mHealth), is a class of assessment techniques that uses multiple daily assessments, often delivered through a smartphone or tablet, to a research participant to gain insight into a variety of states and behaviors at the time that they are experienced in a subject’s natural environment [1]. EMA has become a popular observational study design in the field of public health and provides numerous advantages when compared to traditional retrospective survey methods. The rich, intensive longitudinal data allows for harvesting insights from between-participant variation and within-person variation [1-3]. It minimizes recall bias and increases density in data points, which is especially important for momentary exposures with short induction periods, and it allows for data collection during real-world situations [1,4,5]. In some settings, computerized EMA delivery has replaced paper diary methods because it allows for time-stamped entries and efficient delivery to participant devices that they frequently use in their everyday environments (eg, tablets, smartphones, and computers). This has led to improved adherence to EMA protocols and higher data quality compared with paper diaries [6-11].

### Types of Adherence Considerations

While there are many advantages to electronically delivered EMA, such as frequently measuring momentary events and outcomes, several important adherence-related considerations exist. First, researchers need to be cautious not to overburden participants. Frequent EMA may result in response conditioning and loss of interest in the study, which may significantly hamper adherence if participants experience EMA as intrusive to daily routines (eg, family dinner time or receiving surveys during work commutes) [12-14]. Second, protocol nonadherence (eg, a gap day) is likely nonrandom [11,15] and may be related to momentary exposures that are of interest to the study (eg, when stressors impair the ability of participants to fully engage with the design). Gap days may particularly impair analytic aims concerning across-day, exposure-outcome relationships (eg, rush hour traffic may disrupt meal planning for the following day). Minimizing observation gaps is critical to taking full advantage of EMA methodologies. Third, the characteristics of the study population are important to consider when building an EMA protocol. Young populations may have lower completion rates [16], participants may have complex work commitments that vary over days [17,18] which have been reported in our previous research [18], and respondents may have health conditions that could affect their responsiveness [19].

### Study Design-Related Burden Versus Burden From Momentary Sources

The determinants of EMA adherence are critical to understand because nonadherence could affect the internal and external validity of the study. Low adherence is regularly interpreted as being the result of high participant burden caused by the study design, and measurement of participant burden has been typically characterized using proxy measures specific to the EMA instrument, including survey length, subjective respondent effort, and frequency of instrument delivery [20]. However, there are many factors occurring in a participant’s day-to-day life that may have a direct impact on adherence (eg, momentary high-stress states could affect adherence) [21]. Separating nonadherence due to momentary factors from the burden associated with objective design features may reveal new approaches for anticipating how EMA implementations can be difficult for participants.

This study examines sociodemographic and tempo-spatial correlates of daily participant-reported burden in interacting with an EMA instrument administered in a racially or ethnically diverse and immigrant or refugee sample over an 8-day period. The aims are (1) to examine population characteristics that may be associated with moderate-to-high participant-reported burden and (2) to examine the role of momentary determinants of burden. The study hypothesis associated with the first aim was that burden would be lower among those with both socioeconomic privileges and low momentary and chronic stress. The hypothesis related to the second aim is that burden caused by external or momentary sources, as evidenced by the report of the adults’ experiences of stressors, their mood, and the contextual environments, will present as a transient burden, and that burden caused by the EMA design will present as a high burden on multiple days in a row. The latter case may also indicate that the current study population may have experienced low momentary variability (ie, persistent or disruptive momentary exposures), making less demanding designs more attractive. The results of this study will inform EMA designs for observational studies and ecological momentary intervention studies by helping researchers anticipate what design elements may be appropriate for their target population to maximize momentary exposure and outcome assessment by minimizing participant burden.

## Methods

### Recruitment

The current study draws from Phase 1 (2015-2016) of the Family Matters Study [22], which is a 5-year incremental mixed methods (eg, video-recorded tasks, EMA, interviews, or surveys) study designed to identify novel risk and protective factors for childhood obesity in the home environments of racially or ethnically diverse and primarily low-income children. Phase 1 includes a cross-sectional examination of the family home environment of diverse families (n=150) with children aged between 5 and 7 years each from Black, Hispanic, Hmong, Native American, Somali, and White households (n=25 from each household). Phase 2 is a longitudinal cohort study with parent or child dyads (n=1307; children aged between 5 and 9 years) [22]. Participants were provided with the iPad minis (Apple Inc) by the study to take their EMA surveys and were then given them as an incentive for participating in the study along with US $100 in gift cards.

### EMA Design

EMA was implemented using an iPad mini, on which parents completed signal, event, and end-of-day contingent EMA surveys multiple times throughout the day through a link on the iPad’s home screen in their preferred language (English, Spanish, Somali, or Hmong) [18]. “Signal-contingent surveys” were researcher-initiated and were used in a stratified random manner so that each parent was prompted to fill out a survey 4 times a day within a 3-hour time block (eg, 7 AM-10 AM, 11 AM-2 PM, 3 PM-6 PM, and 7 PM-10 PM) and were set to expire after 1 hour. Each signal-contingent survey was designed to be completed in under 1 minute to minimize response burden and included 10 questions about parent stress and mood, coping self-efficacy, and child eating behaviors and physical activity. “Event-contingent surveys” included 23 questions about a recently eaten meal (eg, who was present, food served, meal atmosphere, child eating behaviors, and food parenting practices) and were either participant-initiated or added to the beginning of a signal-contingent survey to catch any missed event-contingent surveys. Participants were instructed to complete an event-contingent mealtime survey every time they shared a meal with the study child. “End-of-day signal-contingent surveys” were designed to be completed in under 3 minutes and asked 31 questions about parent modeling of eating, physical activity and sedentary behavior, parent stress and mood levels, primary sources of stress, child eating, physical activity and sedentary behaviors, and the caregiver’s overall experience of burden in responding to EMA that day. Participants were given 6 hours to complete the end-of-day survey. Participants were alerted to signal-contingent and end-of-day surveys by an iPad beep or vibration; participants also had the ability to receive a corresponding reminder SMS text message. All EMA responses were time-stamped, and participants were assigned additional days to complete EMA to obtain a minimum of 8 full days of EMA data if they did not meet the minimum EMA responses per day. Based on best practice for EMA, minimum adherence to EMA was defined as submitting 2 signal-contingent, 1 event-contingent mealtime survey, and 1 end-of-day survey, totaling a minimum of 4 surveys per day. Other details regarding the study design unrelated to EMA are described elsewhere [22].

**Figure 1:**
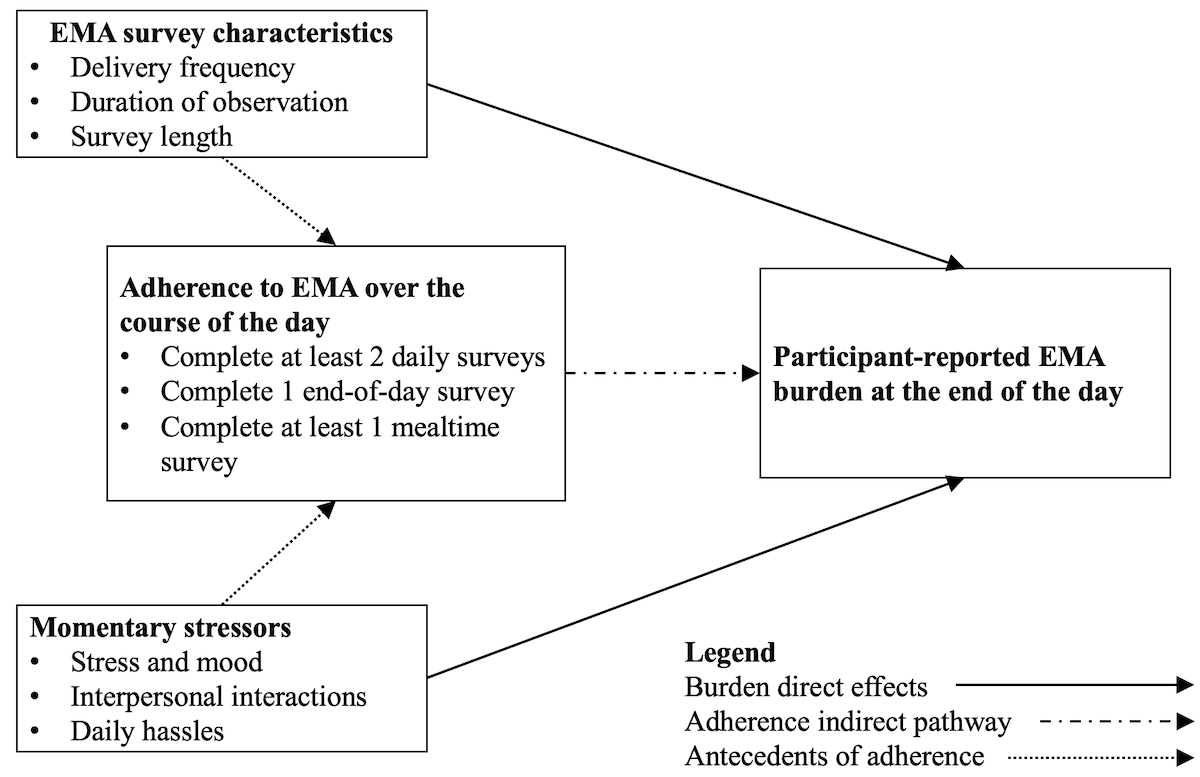
Conceptual model of ecological momentary assessment (EMA) study characteristics and momentary stressors on signal-contingent daily survey adherence and end-of-day participant burden. Sample of person-days restricted to those with at least 1 end of day survey to capture participant burden rating.

An analytic conceptual model is presented in Figure 1 to demonstrate how study design and momentary factors may affect early-day adherence that may be related to measured burden at the end of the day. We are not able to examine the relationship between EMA survey characteristics (different survey lengths or varying delivery frequency) and participant-reported burden because all participants experienced the same surveys (top part of Figure 1). Instead, we estimate the associations between momentary stressors and burden and attempt to identify the extent to which momentary stressors (bottom part of Figure 1), as opposed to survey characteristics, are responsible for participant-reported burden. In addition, we examine adherence and burden across time to assess whether burden is transient, indicating that momentary factors rather than the study design led to burden, versus persistently high burden over multiple days, thus signaling that the study design was burdensome.

### Cross-Sectional Survey Measures

Sociodemographic characteristics of the study population and parent-reported chronic stress were measured by a cross-sectional web-based survey administered after the EMA data collection was complete. These sociodemographic characteristics included caregiver and child age, sex, and objectively-measured anthropometrics (adult BMI and sex- or age-adjusted child BMI percentile), child racial and ethnic group, caregiver nativity (ie, immigrant status), household caregiver structure (single parent with no other adults and with other adults, 2-parent with no other adults and with other adults; 4 categories total), the primary language spoken in the home (English, Spanish, Hmong, or Somali), sources of household income (eg, wages from self, public assistance, wages from other guardians, alimony or child support, and other sources), and average annual household income. Chronic stress was measured by the survey item, “On a scale of 1 to 10, with 1 being not stressed at all and 10 being very stressed, how would you rate your average level of stress in the past 30 days?”

### Participant Burden

The primary study outcome measure was the overall participant-reported burden on the EMA observation day, measured on the end-of-day survey. The item asked, “Overall, how difficult was it for you to fill out the surveys today?” and was measured on a 5-item Likert scale where the increasing burden was measured as not at all, a little, moderately, quite a bit, and extremely. A trait-level participant burden score was computed to evaluate subpopulations that reported low burden (average was less than or equal to “a little”; n=63) and high burden (average was more than “a little”; n=87).

### Adherence to EMA Over the Course of the Day

In the context of this study, nonadherent days were characterized as a completed end-of-day survey on which burden is reported but otherwise did not meet the minimum requirements for an adherent day (at least 2 signal-contingent surveys and at least 1 event-contingent mealtime survey). Each participant responded to at least 8 end-of-day surveys, resulting in 1391 participant-day observations, of which 151 (10.9%) days were nonadherent days. Approximately 65/150 (43%) caregivers in the sample had at least 1 nonadherent day. In some analyses, burden on subsequent days was examined, and 1114 day-pair observations (eg, Monday and Tuesday) of EMA burden were available for analysis. Average days to complete the EMA period (8 per protocol day) were computed for each participant to characterize gap-day frequency.

### Momentary Stressors

Daily stress was measured over the course of the day through signal-contingent surveys (a minimum of 2 and a maximum of 4 each day for an adherent day). On all 151 nonadherent days on which end-of-day burden was measured, participants completed at least 1 daily signal-contingent survey on which momentary stress and mood were reported. Average daily stress (“Overall, how stressful was your day?”) was operationalized as the average of daily surveys on each observation day and could vary on each participant-EMA observation day for the duration of the EMA period. A change score was computed by differencing the stress level on the following and current days to operationalize across-day stress trajectories. Daily depressed mood (“Overall, how SAD or DEPRESSED did you feel today?”) was operationalized in the same way as the caregiver’s average daily stress level, and both measures shared the same Likert scale as the burden outcome. A change score for depressed mood was not examined as a correlate of burden in inferential procedures to avoid multicollinearity with the primary predictor (stress change between days) and to disaggregate possible dependent measurement errors between the 2 variables. Depressed mood on the current day was used as an important control variable in the regression models. Change scores were not possible to compute for subsequent gap days with no daily signal-contingent surveys, resulting in 1392 available participant-day observations to compute the change in daily stress across days. On 1116 day-pairs, 262 (23.5%) days were measured as declining stress, 253 (22.7%) were increasing stress, and 601 (53.9%) had no change in stress. There were 2 day-pairs where stress change was measurable but end-of-day burden was not measured.

### Statistical Analysis

Descriptive statistics were used to examine sociodemographic, stress, and EMA responsiveness correlates of low and high participant burden. A Fisher 2-sided exact statistical test was used for all categorical predictors of high participant burden and equal variance *t* tests were used to examine continuous variables (average EMA burden, average survey reported chronic stress and depressive symptoms, days to complete EMA, parent and child age, and adult and child BMI percentile).

Conditional fixed effects estimators were used to control for all nontime varying confounders and to examine within-caregiver momentary determinants of EMA burden (bottom part of Figure 1), specifically changes in daily stress level, depressed mood, and adherence that day. Adherence was included in the model to examine how partial completion of the EMA protocol may indicate momentary processes were the plausible causes of burden as opposed to study design factors in the case of a negative association. A second model was parameterized to examine how persistent the association is between an adherent day and burden on current and future days, adjusting for weekend versus weekdays. If persistence is high (eg, an adherent day is strongly associated with burden on current and future days), then the burden likely results from study design characteristics rather than momentary factors. A 2-sided statistical significance test was performed at the .05 level for all inferential analyses.

Burden transition probabilities over subsequent EMA observation days were computed to evaluate the population frequency of high-burden states within respondents. High frequency at low burden states and low frequency of persistent high burden over subsequent days were used to interpret if the reported burden was related to momentary stressors as opposed to the EMA instrument. A sensitivity-stratified analysis was computed among the per-protocol adherent caregivers (n=85) and the caregivers with at least 1 nonadherent day (n=65) to evaluate whether daily burden transitions were different for each group. There was no substantive difference in the across-day transition of burden levels for the 2 groups, and the full sample was combined for analysis. All data management and analysis were conducted in Stata (version 17.0 MP; StataCorp).

### Ethical Considerations

The University of Minnesota Institutional Review Board approved Phase 1 of the Family Matters study, and all participants consented in accordance with the Declaration of Helsinki procedures (ID: 1107S02666) [23]. All participant records were deidentified for analysis to ensure participant privacy.

## Results

### Overview

The study population was majority female caregivers and 35 years old and children were 47% (71/150) female and 6.4 years old on average. The majority of parents 57% (85/150) were born in the United States, and immigrant parents (n=62) spoke a language other than English in the home about 65% (40/62) of the time. More than a quarter of households were single-caregiver homes, and 70% (105/150) of the full sample reported less than US $35,000 in annual household income (Table 1 shows participant demographics).

**Table 1:** Phase 1 Family Matters Study demographic and survey characteristics. Stratification by high or low ecological momentary assessment (EMA) burden participants in an ecological momentary study, 2015-2016 (N=150).

Characteristics					None to low levels of EMA burden (n=63)			Moderate to high levels of EMA burden (n=87)			*P* value
**Patient characteristics**											
	EMA burden, mean (SD)			0.4 (0.4)			1.8 (0.5)			<.001^a^	
	Chronic stress over previous 30 days, mean (SD)			3.5 (2.6)			4.3 (2.5)			.04^a^	
	Depressive symptoms in previous 2 weeks, mean (SD)			1.5 (1)			1.4 (0.7)			.65	
	Days to complete EMA, mean (SD)			9.6 (7)			11.8 (8)			.08	
	Female, n (%)			59 (94)			78 (90)			.56	
	Age (years), mean (SD)			34.2 (7.1)			34.7 (7.1)			.66	
	BMI, mean (SD)			30.3 (7.5)			31.3 (7)			.43	
	**Weight status, n (%)**									.74	
		Nonoverweight	16 (25)			19 (22)					
		Overweight	17 (27)			21 (24)					
		Obese	30 (48)			47 (54)					
	Born in the United States, n (%)			43 (68)			42 (48)			.02^a^	
**Child characteristics**											
	Female, n (%)			33 (52)			38 (44)			.32	
	Age in years, mean (SD)			6.4 (0.8)			6.4 (0.8)			.61	
	BMI percentile, mean (SD)			76.5 (23.4)			75.5 (23.1)			.79	
	**Weight status, n (%)**									.86	
		Nonoverweight	32 (51)			45 (52)					
		Overweight	13 (21)			15 (17)					
		Obese	18 (29)			27 (31)					
**Household characteristics**											
	**Household structure, n (%)**									.13	
		1 parent (no other adults)	12 (19)			25 (29)					
		1 parent (with other adults)	6 (10)			12 (14)					
		2 parents (no other adults)	40 (63)			38 (44)					
		2 parents (with other adults)	5 (8)			12 (14)					
	**Family race or ethnicity, n (%)**									.08	
		Black	12 (19)			13 (15)					
		Hispanic	10 (16)			15 (17)					
		Hmong	13 (21)			12 (14)					
		Native American	11 (17)			14 (16)					
		Somali	4 (6)			21 (24)					
		White	13 (21)			12 (14)					
	**Preferred language in home, n (%)**									.04^a^	
		English	49 (78)			56 (64)					
		Spanish	7 (11)			9 (10)					
		Hmong	4 (6)			3 (3)					
		Somali	3 (5)			17 (20)					
		Not reported	0 (0)			2 (2)					
	**Household income (US $), n (%)**									.55	
		<20,000	20 (32)			30 (34)					
		20,000-34,999	23 (37)			32 (37)					
		35,000-49,999	7 (11)			9 (10)					
		50,000-74,999	3 (5)			9 (10)					
		75,000-99,999	4 (6)			3 (3)					
		≥100,000	6 (10)			3 (3)					
		Not reported	0 (0)			1 (1)					

^a^Values are significant at *P*<.05.

The burden assessment is on a 0 to 4 scale, where 0 indicates no reported burden, 2 indicates moderate burden, and 4 indicates extreme burden on each end-of-day EMA assessment. Chronic stress is on a 1-10 scale, where 1 indicates not stressed and 10 indicates very stressed in the past 30 days. Depressive symptoms in the previous 2 weeks were measured on a 4-item Likert scale, where 1 indicated never or rarely and 4 indicated nearly every day feeling down, depressed, or hopeless. Both chronic stress and depressed mood were measured through parent survey self-reports. Family race or ethnicity are self-identified categories that the caregiver best characterized as the identity and culture of household members and the home environment.

### Research Question 1: What are the Sociodemographic Correlates of EMA Burden?

The overall reported EMA burden in the sample was low (Table 1). Parents who reported overall low burden generally reported not at all or a little daily burden of 0.4 (SD 0.4), and those who reported overall moderate-to-high burden generally reported a little burden to moderate daily burden of 1.8 (SD 0.5), which was statistically different at *P*<.001. Parent reports of chronic stress levels over the previous 30 days were elevated among high-burden caregivers with 4.3 (SD 2.5) compared to low-burden caregivers with 3.5 (SD 2.6; *P*=.04). The composition of immigrant caregivers among the high-burden subpopulation was about 20 percentage points higher than the low burden subgroup (*P*=.02), and the English language was less commonly spoken in households where caregivers reported a high overall burden by about 14 percentage points compared to the low burden subgroup (*P*=.04). All other demographic characteristics were not found to be predictive of overall EMA burden, except for wages earned by oneself (*P*=.049; results not presented in Table 1).

### Research Question 2: What are the Momentary Determinants of EMA Burden?

Depressed mood levels and changes in stress were examined as predictors of EMA burden after controlling for survey-day adherence (top panel in Table 2). There was strong evidence that increased stress levels from one day to the next (+0.07 burden; *P*=.008) and within-day depressed mood (+0.15 burden; *P*=.002) were each independently associated with elevated EMA burden.

**Table 2:** Ecological momentary assessment study protocol adherence association with survey burden within and across days and the association between change in stress level and survey burden.

Predictor variable				Burden level β coefficient		95% CIs		*P* value
**Change in daily stress level and burden; conceptual model: momentary antecedents of burden**								
	Change in daily stress level (current day less previous day)			0.07^a^		0.02^a^ to 0.13^a^		.008^a^
	Depressed mood level (current day)			0.15^a^		0.05^a^ to 0.25^a^		.002^a^
	Adherent day (current day)			–0.34^a^		–0.52^a^ to –0.16^a^		<.001^a^
**Participant adherence and burden; conceptual model: survey antecedents of burden**								
	Survey submitted on a compliant day							
		Current-day adherent	–0.39^a^		–0.60^a^ to –0.18^a^		<.001^a^	
		1-day lag adherent	–0.36^a^		–0.56^a^ to –0.16^a^		.001^a^	
		2-day lag adherent	–0.08		–0.28 to 0.13		.47	
	Weekend survey day (reference: weekday)			0.04		–0.08 to 0.16		.51

^a^Values significant at *P*<.05.

Per-protocol adherence was examined as a predictor of burden over 3 days (bottom panel in Table 2). Adherence on the current day was associated with a –0.39 burden (95% CI –0.60 to –0.18; *P*<.001), and current-day adherence was also negatively associated with a next-day –0.36 burden (95% CI –0.56 to –0.16; *P*<.001). This adherence pattern did not extend to the burden reported on the third day (–0.08 burden; *P*=.47), and weekend days were not found to be more or less burdensome (*P*=.51).

Within-participant models for repeated measure data were fitted that control for all time-invariant participant characteristics. Adjustment for time-varying predictors includes weekdays and the composition of participant daily study adherence.

The parent report of survey burden in the current day was –0.39 lower (95% CI –0.60 to –0.18) when the parent was adhering to the study protocol. Adherence was also associated with decreased survey burden on the following day (–0.36; 95% CI –0.56 to –0.16), but the adherence association with reduced burden dissipated by the second day. Weekends were not associated with a higher or lower survey burden compared to weekdays (*P*=.51).

The transition between states of burden was examined in 1114 day-pairs (eg, Monday to Tuesday; Table 3). On 59.4% (662/1114) of days, there was no change in reported burden between days. Over day-pairs, both days were between “none” and “moderate” 82.5% (919/1114) of the time. The subsequent day transitioned from an elevated burden state of “quite a bit” or “extreme” to a lower burden state in 7% (79/1114) of days, and from a low burden state to an elevated burden state in 6.5% (73/1114) of days. Persistently high burden was low, as indicated by only 4% (43/1114) of day-pairs involving “quite a bit” or “extreme” burden on both days.

**Table 3:** Daily transition probabilities and frequency of ecological momentary assessment (EMA) burden over 1114 day-pairs in an EMA study.

	Next-day burden level frequency (overall cell probability %)						
Current-day burden level	None	A little	Moderate	Quite a bit	Extreme	Total (n=1114)	
None	315 (28.3)^a^	44 (4)	58 (5.2)	10 (0.9)	6 (0.5)	433 (38.9)	
A little	44 (4)	89 (8)^a^	48 (4.3)	18 (1.6)	1 (0.1)	200 (18)	
Moderate	52 (4.7)	44 (4)	225 (20.2)^a^	26 (2.3)	12 (1.1)	359 (32.2)	
Quite a bit	7 (0.6)	18 (1.6)	31 (2.8)	19 (1.7)^a^	5 (0.5)	80 (7.2)	
Extreme	8 (0.7)	6 (0.5)	9 (0.8)	5 (0.5)	14 (1.3)	42 (3.8)	

^a^Indicates no change in the state of EMA burden between one day and the next. Cells to the left of these indicate the frequency of days in which burden declined the following day, and cells to the right indicate the frequency of increasing burden days.

Interpretation example: Participants were required to submit at least 2 daily surveys, at least 1 mealtime survey, and 1 final survey in which the overall EMA burden was measured. On 59.4% (662/1114) of days, there was no change in reported burden between days, and over day-pairs, both days were between “none” and “moderate” 82.5% (919/1114) of the time. The subsequent day transitioned from an elevated burden state of “quite a bit” or “extreme” to a lower burden state in 7% (79/1114) of days and from a low burden state to an elevated burden state in 6.5% (73/1114) of days. Persistently high burden was low, as indicated by only 4% (43/1114) of day-pairs involving “quite a bit” or “extreme” burden on both days.

## Discussion

### Overview

Findings from the current study overall suggest that the current EMA study was tailored to a racially or ethnically diverse and immigrant or refugee study population, and momentary processes were sufficiently captured (ie, stress responses) over a 1-week study duration. Specifically, there was no evidence that participants reported persistently high levels of burden that would indicate the EMA design elements for this study were burdensome for caregivers (research aim 1). Across-day variability in study burden indicated that human-environment interactions (eg, response to sources of stress) rather than human-instrument interactions (eg, frequency of daily survey delivery) were related to participant burden, suggesting that comparable protocols are appropriate for examination of momentary states in low-income, racially or ethnically diverse, and immigrant or refugee sample populations (research aim 2). There was also evidence that some subpopulations may have trouble adhering to EMA studies. Participant characteristics that should be considered include chronic stress levels, immigrant status, and the language primarily spoken in the home—specifically non-English speaking households.

### Importance of Population Characteristics for Protocol Development

Study population attributes continue to be important for the effective deployment of mHealth observational study designs. This study’s results support and extend previous studies that examined smartphone-based data collection among populations with affective disorders [24] and with substance-use dependency [25] in that mHealth studies interested in the relationship between momentary states and health and health behavior outcomes are feasible and pose low burden to participants. New findings from this study showed that factors affecting adherence to study protocol may include the spoken language of participants, indicating the critical importance of instruments that are tailored to the primary or preferred language of respondents, as well as the levels of strain and chronic stressors that a study population may experience during the observation period. Word recognition, for example, has been identified as an important determinant of study adherence in other studies [26]. Although measures were translated for families who spoke a language other than English, immigrant families experienced a higher burden, suggesting that other factors and chronic stressors unique to this subsample, such as acculturative stress, may influence adherence to protocols [27]. With respect to chronically stressed populations, research that addresses the consequences of momentary stress on within- and across-day behavioral- and health-related relationships should pay careful attention to longitudinal patterns of stress level and other measures of psychological distress (eg, depressive symptoms) that may affect measurement and missingness. For example, in a previous study, populations with substance-use dependency were less adherent than nondependent samples, which can affect both the internal validity and representativeness of findings for other comparable groups [25]. Cultural barriers and a lack of overall trust in health research may affect patterns of missingness in EMA data that could further affect the representativeness of study data on the intended population [28,29]. In this study, similar adherence patterns were found among chronically stressed populations compared to less chronically stressed respondents. There was little evidence of persistent, high burden, suggesting that some groups may experience stressors at greater intensity and frequency than others; further, the second-day lag, nonadherence-burden association was observed to dissipate as a predictor of subsequent burden 2 days later. This reality is why EMA can be an effective tool for describing momentary human-environment interactions.

In this study, the length of surveys, study duration, and frequency of EMA assessments were intentionally limited to minimize participant burden, and direct measurement of burden supported that these design decisions were effective. Other studies have made different decisions about these design factors and had lower adherence and loss of follow-up. For example, a 6-month study of food-related behaviors using a smartphone data collection methodology found that adherence declined over the course of the study [30]. Waving EMA observation intervals to allow for resting states (eg, 1 week on, 1 week off, and then 1 week on again for a 2-wave design) may be one effective strategy to improve confidence in measurement validity, long-term retention of participants, and overall adherence in EMA studies. Long study duration and high sampling frequency were related to poor data quality in a study [31], and in another study, a 10-week observation period found a similar decline in adherence over time that was speculated to indicate a waning interest in the study [32]. In the latter study, adherence improved following direct interaction with providers and phone contact with staff during the scheduling of follow-up visits. Attentive support from the research team may buffer against participant burden and increase adherence to the study protocol.

### Momentary and Study-Related Stress and Burden

Direct measurement of study burden is an important tool for assessing the feasibility of design elements that may be used in future studies. Although proxy measures of burden, including time to complete EMA and time to start a survey following delivery of a survey notification, can be effective in assessing measurement validity, more research is needed to assess how respondents interact with the data collection instrument. In this study, the receipt of participant feedback at the end of the day was more effective in quantifying patterns of burden that may be related to momentary factors, including stress sources and the quality of caregiver-child interactions, than the study design itself, which improves the replicability of results in future implementations. As observed in this study, measured levels of low-to-moderate burden may be good indicators that respondents are paying careful attention to survey items in balance with their other environmental demands. The daily participant-reported burden was also effective in describing state versus trait patterns of subpopulations in this study. Evidence of varied states of burden indicated that momentary processes were captured during the 1-week observation period. The low frequency of persistent high burden indicated that this study was reasonably tailored to the needs of a predominantly low-income, racially or ethnically diverse and immigrant or refugee population.

### Strengths and Limitations

This study had notable strengths, including stratified recruitment of a racially or ethnically diverse and immigrant or refugee study population and instruments translated into participants’ preferred language. Although incentives were given for a complete 8 days of data collection, the daily completeness of the protocol was not incentivized in this implementation. Bonus incentives for consecutive-day adherence may increase the quality of data collected over the course of the study period, as consecutive-day data allows for better evaluation of real-time influences on outcomes across days. The greater the frequency of nonsequential EMA reporting, the greater was possibility that selection bias may affect the representativeness and transportability of study findings to other populations. Bonus incentives may also be critical for minimizing burden and promoting adherence in EMA studies that are carried out over long time frames. A request for participant feedback about their experience of burden increased confidence about differentiating between momentary and study-related burden. While the 8-day observation period was adequate for characterizing the low study burden for the current protocol, future studies may require longer observation intervals to capture rare momentary exposures (eg, periodic financial stressors or housing insecurity) for significant life events. These studies should measure participant burden to determine if these findings cohere for long-term observation periods. Replication should also be performed in larger study samples to determine if comparable protocols represent typical respondent experiences.

### Conclusions

The EMA study burden was found to be low in an 8-day EMA study of the momentary sources of stress, depressed mood, and caregiver-child interactions in a racially or ethnically diverse and immigrant or refugee sample of adult caregivers. Attention to the sociodemographic attributes of the sample, including chronic stress levels, nativity, and preferred language, is important for minimizing participant burden and improving data quality. Momentary stress is a strong determinant of participant-experienced burden and may affect adherence to mHealth study protocols. EMA stands to be an important observational design for providing critically relevant insight into human-environment interactions that advances intervention development appropriate for dynamic public health challenges when the design is carefully tailored to the study population and research objectives.

## Abbreviations

EMA: ecological momentary assessment
mHealth: mobile health
